# Molecular alterations in skeletal muscle in rheumatoid arthritis are related to disease activity, physical inactivity, and disability

**DOI:** 10.1186/s13075-016-1215-7

**Published:** 2017-01-23

**Authors:** Kim M. Huffman, Ryan Jessee, Brian Andonian, Brittany N. Davis, Rachel Narowski, Janet L. Huebner, Virginia B. Kraus, Julie McCracken, Brian F. Gilmore, K. Noelle Tune, Milton Campbell, Timothy R. Koves, Deborah M. Muoio, Monica J. Hubal, William E. Kraus

**Affiliations:** 1 Department of Medicine, Duke Molecular Physiology Institute, Duke School of Medicine, Durham, NC USA; 2 Biomedical Engineering, Duke University, Durham, NC USA; 3 Harvard University School of Law, Boston, MA USA; 4 Department of Surgery, Duke School of Medicine, Durham, NC USA; 5 Department of Emergency Medicine, Indiana University, Indianapolis, IN USA; 6 George Washington University, Washington, DC USA

**Keywords:** Gene expression, Metabolomics, Satellite cells, Fibrosis, Inflammation

## Abstract

**Background:**

To identify molecular alterations in skeletal muscle in rheumatoid arthritis (RA) that may contribute to ongoing disability in RA.

**Methods:**

Persons with seropositive or erosive RA (*n* = 51) and control subjects matched for age, gender, race, body mass index (BMI), and physical activity (*n* = 51) underwent assessment of disease activity, disability, pain, physical activity and thigh muscle biopsies. Muscle tissue was used for measurement of pro-inflammatory markers, transcriptomics, and comprehensive profiling of metabolic intermediates. Groups were compared using mixed models. Bivariate associations were assessed with Spearman correlation.

**Results:**

Compared to controls, patients with RA had 75% greater muscle concentrations of IL-6 protein (*p* = 0.006). In patients with RA, muscle concentrations of inflammatory markers were positively associated (*p* < 0.05 for all) with disease activity (IL-1β, IL-8), disability (IL-1β, IL-6), pain (IL-1β, TNF-α, toll-like receptor (TLR)-4), and physical inactivity (IL-1β, IL-6). Muscle cytokines were not related to corresponding systemic cytokines. Prominent among the gene sets differentially expressed in muscles in RA versus controls were those involved in skeletal muscle repair processes and glycolytic metabolism. Metabolic profiling revealed 46% higher concentrations of pyruvate in muscle in RA (*p* < 0.05), and strong positive correlation between levels of amino acids involved in fibrosis (arginine, ornithine, proline, and glycine) and disability (*p* < 0.05).

**Conclusion:**

RA is accompanied by broad-ranging molecular alterations in skeletal muscle. Analysis of inflammatory markers, gene expression, and metabolic intermediates linked disease-related disruptions in muscle inflammatory signaling, remodeling, and metabolic programming to physical inactivity and disability. Thus, skeletal muscle dysfunction might contribute to a viscous cycle of RA disease activity, physical inactivity, and disability.

**Electronic supplementary material:**

The online version of this article (doi:10.1186/s13075-016-1215-7) contains supplementary material, which is available to authorized users.

## Background

Despite a vast array of pharmacologic agents available to treat rheumatoid arthritis (RA), management is often complicated by insufficient treatment response, drug toxicity and contraindications, poor access to care and/or medications, and/or damage that predates medical intervention. These barriers lead to or are accompanied by systemic manifestations, disease-associated co-morbidities, chronic pain, physical inactivity, dysmobility, and poor physical function. Thus, further advances in RA care require identification of factors contributing to persistent deficiencies in quality of life and physical function, despite access to excellent anti-rheumatic medications.

Importantly, inactivity and muscle wasting are two important contributors to RA-related morbidity and mortality. Approximately half of patients with RA do not perform even a single bout of weekly physical exercise [1]. The sedentary lifestyle common to patients with RA gives rise to physical deconditioning and muscle atrophy, both of which are associated with osteoporosis, impaired immune function, glucose intolerance, insulin resistance, loss of independence, and increased mortality [2].

In addition to physical inactivity, other factors that likewise promote muscle loss and disability in patients with RA include inadequate protein ingestion, glucocorticoid treatment, and pro-inflammatory cytokines, all resulting in reduced myocyte protein synthesis and increased protein degradation [2, 3]. Inflammation can impact normal muscle turnover and responses to injury, both of which require an exquisitely coordinated remodeling process involving activation, proliferation and differentiation of muscle stem cells—also known as satellite cells. These processes are mediated largely by signals from intramuscular immune cells: neutrophils, regulatory T cells, pro-inflammatory M1 macrophages, and anti-inflammatory M2 macrophages.

The established roles of inflammation in both skeletal muscle remodeling and RA pathophysiology raise obvious questions regarding the potential interplay between muscle dysfunction and RA morbidity. Whereas the link between pro-inflammatory cytokines and muscle dysfunction has been investigated intensely in the context of diseases such as diabetes and cancer cachexia, this topic has remained surprisingly unexplored in RA. In the current study we sought to identify molecular perturbations in muscle specimens from individuals with RA, and to test the hypothesis that skeletal muscle inflammatory markers and derangements in tissue remodeling might contribute to metabolic decline and disability in these patients. Herein, we report that disease-activity-related muscle inflammatory markers are related to physical inactivity, and moreover, that disrupted skeletal muscle repair processes are associated with greater disability. These findings support a model in which skeletal muscle deterioration contributes to a vicious cycle of disease activity, muscle inflammatory signaling and disrupted remodeling, physical inactivity, and disability in patients with RA.

## Methods

### Design and participants

This was a cross-sectional investigation of individuals with RA and matched controls collected from the Durham, NC area. The RA group met the following criteria: (1) RA diagnosis meeting American College of Rheumatology 1987 criteria [4]; (2) seropositive disease (positive rheumatoid factor or anti-cyclic citrullinated peptide) or evidence of erosions on hand or foot imaging; (3) no medication changes within the three months prior to study enrollment; and (4) daily prednisone use ≤5 mg. Healthy participants without a diagnosis of RA, without joint pain, and without joint swelling lasting more than a week were matched to individual participants with RA by gender, race, age within 3 years, and body mass index (BMI) within 3 kg/m^2^. Exclusions included current pregnancy, type 2 diabetes mellitus, and known coronary artery disease. Further specific details on questionnaires and measurement protocols have previously been described [5]. This study was in compliance with the Helsinki Declaration and was approved by the Duke University Institutional Review Board.

Assessments of both groups included questionnaires, physical exams for disease status, fasting blood collection, intravenous glucose tolerance tests for insulin sensitivity, 7 days of accelerometer-measured physical activity, computed tomography (CT) imaging of abdomen and thigh, and *vastus lateralis* muscle biopsies [5]. Disability (health assessment questionnaire-disability index (HAQ-DI) and co-morbidities (co-morbidity index) were assessed by previously published questionnaires [6, 7]. Disease activity assessed by the disease activity score in 28 joints (DAS-28) was determined from a patient-completed visual analog scale, physician-determined numbers of tender and swollen joints, and erythrocyte sedimentation rate [8]. Plasma concentrations of inflammatory markers and cytokines were determined by immunoassay [5] and nuclear magnetic resonance (NMR) spectroscopy (GlycA) [9]. Insulin sensitivity was determined using Bergman’s minimal model [10] and concentrations of glucose and insulin (glucose: Beckman-CoulterDXC600; insulin: electrochemiluminscent assay from Meso Scale Discovery) at each of 29 time points during the intravenous glucose tolerance test.

Physical activity was measured with 7 days of accelerometry. After completing assessments, accelerometers (RT3, Stayhealthy, Inc., Monrovia, CA, USA) were provided to participants. Participants also received a pre-addressed and postage-applied box for return and directions for wearing on the waist above the right knee during waking hours for 7 days. Accelerometer data were evaluated for validity and non-wear time, and categorized into metabolic equivalents (METs) as previously described [11]. After data cleaning, valid data were available for 41 persons with RA and 31 controls. Time spent exercising was defined as the sum of time spent performing activity at METs equal to or greater than 3. CT scan analyses were performed using OsiriX (Pixmeo) to determine adipose and muscle tissue size and muscle tissue density (greater tissue density is indicative of less inter-muscular adipose tissue) [5]. Standard Bergstrom needle muscle biopsies were performed on the *vastus lateralis* in the fasting state; participants consumed only water during the 12 hours overnight prior to the biopsy [12]. Tissue was flash frozen in liquid nitrogen and stored at −80 ° C.

### Skeletal muscle inflammatory marker measurements

Flash frozen muscle samples (5–10 mg) were homogenized in a buffer consisting of 1% Nonidet-P40, 1 mM EDTA, 150 mM NaCl, and 20 mM Tris-Cl for ELISA-based measures of muscle (m) interleukin (IL)-1β, mIL-6, mIL-8, m-tumor necrosis factor (TNF)-α (MSD 4-plex; K15053D-1) and m-toll like receptor (TLR)-4 (Abnova; KA1238). Assays were performed according to the manufacturers’ directions except for the addition of a 30-minute, room temperature, blocking step with 5% BSA followed by three PBS-T washes. Concentrations were normalized to starting masses. Spike-and-recovery assays for all analytes achieved 80–100% recovery confirming lack of assay interference by muscle homogenates. For each cytokine, the mean intra-assay and inter-assay coefficients of variation were: mIL-1β 8.5%, 13.2%; mIL-6 3.5%, 1.5%; mIL-8 4.0%, 4.0%; mTNF-α 8.4%, 10.4%; and mTLR-4 1.7% (only one plate was used for analyses).

### Gene expression analyses

Muscle samples were selected for gene expression analyses in an effort to span the range of RA disease activity seen in the larger sample; these corresponded to the following DAS-28 categories: remission (*n* = 7), low (*n* = 4), moderate (*n* = 6), and high activity (*n* = 3). For each RA muscle sample, the corresponding sample from a control matched by age, gender, and BMI was included.

For RNA preparation, flash frozen muscle samples (20–30 mg) were homogenized in 1 mL TRIzol® (Thermo Fisher Scientific, Inc, Waltham, MA, USA). Biotinylated total RNA was generated using the Illumina TotalPrep RNA amplification kit (Life Technologies, Grand Island, NY, USA); 200 nanograms of RNA were used for the kit. The quality of the RNA was determined using the Bioanalyzer RNA Nano chip assay (Agilent, Santa Clara, CA, USA). Quantification of the RNA was determined using the Quant-iT RiboGreen RNA Assay Kit. The Human HT-12v3 Expression BeadChip (Illumina, San Diego, CA, USA) was used for quantitative whole genome RNA profiling. Biotinylated RNA (750 ng) was hybridized to the BeadChip and washed; Cy3-SA was then introduced to the hybridized samples and the BeadChips scanned on the Illumina iScan system according to manufacturer’s protocol. Quality control was performed using the Illumina GenomeStudio tools.

Gene expression fold-differences between groups were compared in Partek Genomics Suite (Partek, Inc.; St. Louis, MO, USA). For pathway analyses, differentially expressed genes (*p* < 0.02) were evaluated using the Ingenuity Pathway Analysis software (IPA, www.ingenuity.com). IPA identified the canonical pathways containing the greatest number of significant, differentially expressed genes in the dataset. IPA also generated novel networks of related genes and molecules based on the relationships present in the current literature.

### Skeletal muscle metabolic intermediate measurements

Metabolites were measured in muscle from all participants (n = 102). Flash frozen muscle biopsies weighing approximately 25 mg were diluted 20 times (wt:vol) in ice-cold 50% acetonitrile containing 0.3% formate and homogenized for 120 sec in a TissueLyser II (Qiagen) at 30 Hz. Amino acids, organic acids, and acylcarnitines were analyzed using stable isotope dilution techniques in the Duke Molecular Physiology Metabolomics Core. Amino acids and acylcarnitine measurements were made by flow injection tandem mass spectrometry (MS) as previously described [13, 14]. The data were acquired using a Micromass Quattro Micro liquid chromatography (LC)-MS system running MassLynx 4.0 software (Waters Corporation, Milford, MA, USA). Organic acids were quantified using methods described previously [15] employing Trace Ultra GC coupled to ISQ MS operating under Xcalibur 2.2 (Thermo Fisher Scientific, Austin, TX, USA). All data are expressed as picomoles/mg tissue.

### Statistical analyses

Accounting for the repeated measures in matched participants, patients with RA and controls were compared using mixed models. Muscle inflammatory molecules and metabolic intermediates were logarithmically transformed prior to group comparisons. Bivariate associations were assessed with Spearman correlation. Gene expression fold-changes were compared in Partek using analysis of variance (ANOVA). All other statistical analyses were performed using SAS 9.4 (SAS, Cary, NC). All data are available from the corresponding author upon reasonable request.

## Results

### Clinical measures and skeletal muscle inflammatory markers

As shown in Table 1, persons with RA were well-matched to controls by age, gender, and BMI. Patients with RA were recruited based on the inclusion criteria described and without respect to physical activity levels, body mass or body composition; similarly controls were included upon matching a patient with RA by age, gender, and BMI. Despite this, patients with RA and controls were similar with respect to physical activity levels, abdominal and thigh adipose depot size, muscle area, and muscle density [5, 11]. In those with RA, there was more co-morbidity, disability, and systemic inflammation; specifically, greater serum concentrations of high sensitivity C-reactive protein (hs-CRP), IL-6, and TNF-α (*p* < 0.05 for all) [5]. When skeletal muscle inflammatory markers were compared, there was approximately two times greater concentrations of the muscle cytokines, mIL-6 (*p* = 0.006) and mIL-8 in RA (*p* = 0.059) (Table 1).

**Table 1:** Participant characteristics

Variable	All participants (*n* = 102)	Rheumatoid arthritis (*n* = 51)	Matched controls (*n* = 51)
Age (years)	54.2 (12.5)	54.8 (13.2)	53.8 (11.9)
BMI (kg/m^2^)	30.0 (6.4)	30.3 (7.5)	29.6 (5.1)
Waist circumference (cm)	94.1 (15.2)	94.9 (16.8)	92.9 (13.3)
Race			
Caucasian	74 (72.6%)	36 (70.6%)	38 (74.5%)
African American	27 (26.5%)	14 (27.5%)	13 (25.5%)
Pacific Islander	1 (1.0%)	1 (2.0%)	0
Gender			
Female	72 (70.6%)	36 (70.6%)	36 (70.6%)
Male	30 (29.4%)	15 (29.4%)	15 (29.4%)
Physical activity (kCal/day)	557.1 (280.8)	517.7 (279.4)	609.1 (278.7)
Physical activity (MET-hr/day)	5.4 (2.6)	4.9 (2.5)	6.0 (2.5)
Disease duration (months)	NA	138.9 (136.3)	NA
HAQ-disability index	0.46 (0.6)	0.68 (0.7)^*^	0.00 (0.0)
Comorbidity index	1.2 (1.2)	1.6 (1.1)^*^	0.6 (0.9)
DAS-28 mean (SD)	NA	3.0 (1.4)	NA
Remission (DAS-28 < 2.6)		19 (40%)	
Low activity (DAS-28 2.6‒3.2)		8 (17%)	
Moderate activity (DAS-28 3.2‒5.1)		16 (33%)	
High activity (DAS-28 > 5.1)		5 (10%)	
Rheumatoid factor positive	NA	42/47 (89.4%)	NA
Anti-cyclic citrullinated antibody positive	NA	21/22 (95.6%)	NA
Erosions present on radiographs	NA	21/38 (55.2%)	NA
Medication use	NA		
Etanercept		10 (19.6%)	NA
Infliximab		2 (3.9%)	NA
Adalimumab		5 (9.8%)	NA
Abatacept		5 (9.8%)	NA
Methotrexate		39 (76.5%)	NA
Leflunomide		1 (2.0%)	NA
Sulfasalazine		0	NA
Hydroxychloroquine		10 (19.6%)	NA
Nonsteroidal anti-inflammatory agents		18 (35.3%)^*^	1 (4.0%)
Prednisone (<5.0 mg/day)		13 (25.5%)	NA
Systemic inflammation			
hsCRP (mg/L)	3.0 (3.9)	3.7 (4.9)^*^	2.4 (2.9)
IL-1beta (pg/mL)	0.23 (5.3)	0.22 (4.1)	0.17 (6.4)
IL-6 (pg/mL)	4.9 (2.8)	8.9 (2.9)^*^	2.7 (1.6)
IL-8 (pg/mL)	8.2 (2.1)	8.9 (1.8)	7.5 (2.3)
TNF-alpha (pg/mL)	13.7 (2.3)	19.9 (2.4)^*^	9.5 (1.7)
IL-18 (pg/mL)	408.3 (1.4)	440.6 (1.3)	379.3 (1.4)
Adiposity and muscle tissue			
Abdominal total adipose area (cm^2^)	427.9 (181.0)	408.4 (199.5)	447.3 (160.2)
Abdominal subcutaneous adiposity (cm^2^)	303.3 (143.7)	304.5 (154.2)	302.1 (133.9)
Abdominal visceral adiposity (cm^2^)	124.6 (93.2)	104.0 (77.1)^*^	145.2 (103.6)
Abdominal liver density (Hu)	59.0 (11.6)	59.7 (10.6)	58.2 (12.9)
Thigh total area (cm^2^)	249.6 (65.4)	248.8 (73.6)	251.7 (57.1)
Thigh total adipose area (cm^2^)	250.2 (66.0)	134.3 (65.8)	110.9 (68.0)
Thigh subcutaneous adiposity (cm^2^)	122.6 (67.6)	122.6 (62.7)	113.8 (54.0)
Thigh inter-muscular adiposity (cm^2^)	11.3 (7.4)	11.7 (6.7)	11.0 (8.1)
Thigh muscle area (cm^2^)	119.6 (35.1)	114.5 (37.1)	125.4 (32.1)
Thigh muscle density (Hu)	54.0 (8.1)	50.7 (6.2)	55.4 (6.8)
Skeletal muscle inflammatory markers			
IL-1β (pg/mL/mg)	0.035 (0.084)	0.037 (0.093)	0.033 (0.069)
IL-6 (pg/mL/mg)	0.012 (0.010)	0.014 (0.010)^*^	0.008 (0.007)
IL-8 (pg/mL/mg)	0.139 (0.178)	0.169 (0.211)	0.097 (0.106)
TNF-α (pg/mL/mg)	0.012 (0.015)	0.014 (0.016)	0.010 (0.014)
TLR4 (pg/mL/mg)	0.891 (0.666)	0.859 (0.692)	0.937 (0.625)

Data are presented as means (SD) for continuous variables and number (percentages) of participants for dichotomous variables. Data that were not normally distributed (systemic inflammatory markers and cytokines) are presented as geometric means (SD). Physical activity data reflect rheumatoid arthritis (RA) (n = 41) and controls (n = 31) with valid data. *BMI* body mass index, *MET* metabolic equivalents, *HAQ* health assessment questionnaire, *DAS-28* disease activity score with 28-joint count, *hsCRP* high sensitivity C-reactive protein, *IL* interleukin, *TNF* tumor necrosis factor, *Hu* Houndsfield units, *TLR* toll-like receptor

^*^ *p* < 0.05 for comparison with matched controls

Akin to disease activity, RA muscle inflammatory markers exhibited variation across a broad range (Table 1). Muscle inflammatory marker concentrations were positively associated with disease activity (mIL-1β, mIL-8), disability (mIL-1β, mIL-6), and pain (mIL-1β, mTNF-α, mTLR-4) (*p* < 0.05 for all) (Table 2). Muscle cytokines, mIL-1β and mIL-8, were negatively correlated with use of biological agents; mTNF-α was negatively correlated with use of non-biological disease-modifying therapy (*p* < 0.05 for all) (Table 2). Importantly, there were no correlation between muscle inflammatory marker concentrations and prednisone treatment.

**Table 2:** Skeletal muscle inflammatory marker correlations in patients with rheumatoid arthritis

Variable	Muscle IL-6	Muscle IL-8	Muscle TNF-α	Muscle IL-1β	Muscle TLR-4
Age (years)	−0.07	0.05	0.09	−0.09	**−0.29** ^*^
BMI (kg/m^2^)	0.24	−0.05	−0.23	−0.10	−0.25
Disease activity (DAS28)	0.23	**0.30** ^*^	0.14	**0.35** ^*^	−0.01
Disability (HAQ-DI)	**0.33** ^*^	0.19	0.09	**0.33** ^*^	0.12
Pain (VAS)	0.15	0.17	**0.29** ^*^	**0.39** ^*^	**0.47** ^*^
Prednisone use (yes = 1)	0.14	−0.05	0.00	0.01	−0.01
DMARD use (yes = 1)	−0.04	−0.07	**−0.30**	0.21	0.08
Biologic use (yes = 1)	−0.25	**−0.37** ^*^	0.21	**−0.33** ^*^	0.01
Comorbidity index	0.17	0.12	0.17	0.26	−0.08
Plasma hsCRP (mg/L)	0.20	0.07	0.11	−0.03	−0.17
Plasma IL-1β (pg/mL)	0.01	−0.07	−0.07	−0.14	−0.12
Plasma IL-6 (pg/mL)	−0.03	0.11	0.12	−0.01	−0.10
Plasma IL-8 (pg/mL)	−0.11	0.06	0.11	0.14	0.02
Plasma TNF-α (pg/mL)	**−0.37** ^*^	−0.15	0.02	−0.23	−0.08
Plasma IL-18 (pg/mL)	−0.08	−0.12	−0.02	−0.24	0.06
GlycA (μmol/L)	**0.41** ^*^	**0.38** ^*^	−0.06	0.07	−0.21
HOMA	0.11	0.04	−0.06	−0.10	−0.07
Insulin sensitivity (10^-5.^min^-1^/(pmol/L))	−0.20	−0.19	−0.06	−0.09	−0.18
Fasting insulin (mU/L)	0.13	0.09	−0.13	−0.06	−0.06
Visceral adiposity (cm^2^)	0.11	0.01	−0.28	0.03	−0.23
Abdominal subcutaneous adiposity (cm^2^)	0.21	0.06	−0.19	−0.06	−0.19
Total abdominal adiposity (cm^2^)	0.19	0.07	−0.24	−0.06	−0.28
Thigh muscle density (Hu)	−0.04	−0.10	0.06	0.16	**0.28** ^*^
Thigh inter-muscular adiposity (cm^2^)	0.12	0.01	−0.08	−0.11	−0.12
Thigh subcutaneous adiposity (cm^2^)	**0.31** ^*^	−0.07	−0.11	−0.09	−0.11
Exercise (min/day)	**−0.40** ^*^	**−0.38** ^*^	−0.05	**−0.38** ^*^	−0.11
Physical activity (MET h/day)	**−0.33** ^*^	−0.26	0.10	**−0.35** ^*^	−0.15

Data are shown as Spearman correlation coefficients. *BMI* body mass index, *DAS-28* disease activity score with 28 joint count, *HAQ-DI* health assessment questionnaire disability index, *VAS* visual analog scale, *DMARD* disease-modifying anti-rheumatic drug (methotrexate, leflunomide, hydroxychlorquie), *biologic* biologic DMARD (adalimumab, etanercept, infliximab, abatacept), *hsCRP* high-sensitivity C-reactive protein, *IL* interleukin, *TNF* tumor necrosis factor, *HOMA* homeostasis model assessment, *Hu* Houndsfield units, *MET* metabolic equivalent, *TLR* toll-like receptor. ^*^ *p* < 0.05 for Spearman correlation

In addition to disease-related factors, muscle cytokine concentrations (mIL-1β, mIL-6, and mIL-8) were negatively associated with exercise minutes (*p* < 0.05 for all) (Table 2). Higher mIL-1β and mIL-6 concentrations were associated with less total physical activity (total METs; *p* < 0.05 for both) (Table 2). Although, both mIL-6 and mIL-8 were positively correlated with the systemic inflammatory marker, GlycA (*p* < 0.05 for both) (Table 2), muscle inflammatory marker concentrations were not related to insulin sensitivity or systemic cytokine concentrations.

### Skeletal muscle gene expression

To better understand the etiology of RA muscle inflammatory markers, we compared RA (*n* = 20) and control (*n* = 20) skeletal muscle gene expression: 1939 genes were significantly upregulated or downregulated in RA samples (*p* < 0.05); 445 genes were identified when using a more stringent definition of significance (*p* < 0.02).

To identify other relationships between differentially expressed RA muscle genes, pathway analyses were performed using IPA, which has thousands of canonical pathways onto which our experimental gene expression differences were overlaid. Of those canonical pathways, IPA identified several pathways impacted by differential gene expression in muscle in RA (*p* < 0.05) (Table 3). Except for glycolysis and methionine degradation, these canonical pathways were identified because of reduced RA muscle gene expression for nuclear factor (NF)-kβ2, both nuclear factor of activated T cells (NFAT)5 and NFATC4, or all three. Also, none of the canonical pathways was predicted to be activated or inhibited by gene expression differences in muscle in RA (*Z*-scores < |2|) (Table 3) [16].

**Table 3:** Canonical pathways implicated in gene expression in muscle in rheumatoid arthritis

Pathway	Dataset genes^a^ in pathway (*n*)	Total genes in pathway (*n*)	*Z*-score	*p* value
Wnt/Ca + pathway	5	55	0	0.006
Netrin signaling	4	39	NaN	0.008
Glycolysis	3	24	NaN	0.013
Atherosclerosis signaling	7	121	NaN	0.013
Altered T and B cell signaling in rheumatoid arthritis	5	81	NaN	0.023
Methionine degradation to homocysteine	2	16	NaN	0.043
PI3K signaling in B lymphocytes	6	123	−0.816	0.043
April mediated signaling	3	38	NaN	0.044
B cell activating factor signaling	3	40	NaN	0.049

^a^Dataset genes were those differentially expressed between 20 patients with rheumatoid arthritis and 20 age, gender, and body mass index matched controls (*p* < 0.02). *NaN* Not a number

**Fig. 1:**
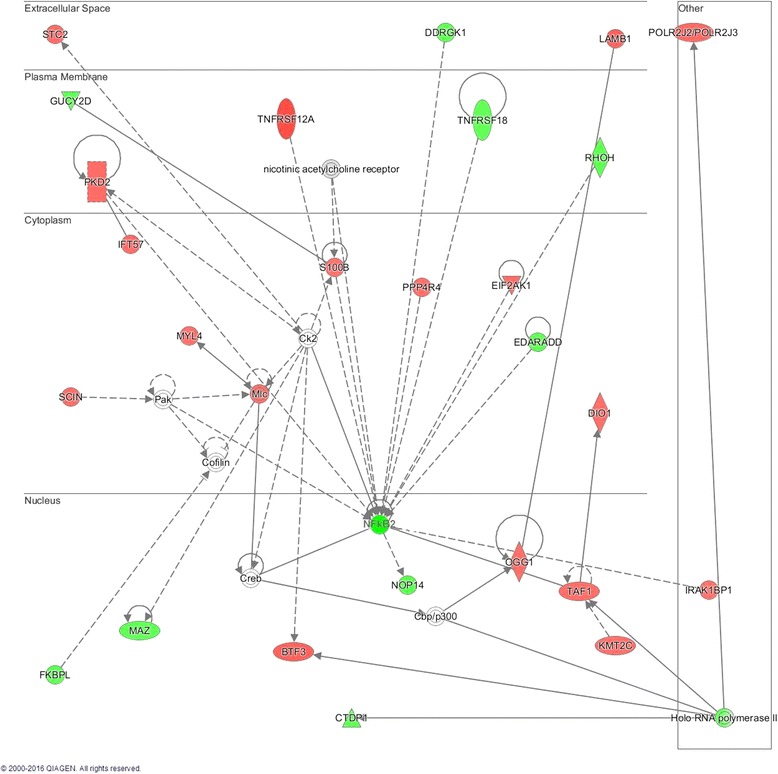
Novel network identified by muscle gene expression in rheumatoid arthritis (RA): gene expression was determined in muscle from 20 persons with RA and 20 controls matched by age, gender, and body mass index. The network shows connections between genes with differential expression in RA relative to control muscle. Genes in *red* were upregulated and genes in *green* were downregulated in muscle in RA

In addition to canonical networks, pathway analyses generate novel networks connecting differentially regulated molecules based on published associations. The IPA-generated novel network with the highest connection score depicted significant differences in expression of genes associated with connective tissue, dental, and dermatological diseases (Fig. 1; Table 4). The prominent pathway connections in muscle in RA were centered on regulation of the NF-kB complex, specifically NF-kB2. These were in the setting of differential regulation of genes in muscle repair and glycolysis.

**Table 4:** Novel network genes

Gene ID	Gene name	RA vs. CONTROL	
		Fold change	*p* value
BTF3	Basic transcription factor 3	1.11	0.003
CTDP1	CTD (carboxy-terminal domain, RNA polymerase II, polypeptide A) phosphatase, subunit 1	−1.04	0.006
DDRGK1	DDRGK domain containing 1	−1.07	0.02
DIO1	Deiodinase, iodothyronine, type I	1.03	0.005
EDARADD	EDAR-associated death domain	−1.06	0.007
EIF2AK1	Eukaryotic translation initiation factor 2-alpha kinase 1	1.05	0.007
FKBPL	FK506 binding protein like	−1.06	0.003
GUCY2D	Guanylate cyclase 2D, membrane (retina-specific)	−1.04	0.004
IFT57	Intraflagellar transport 57	1.04	0.01
IRAK1BP1	Interleukin-1 receptor-associated kinase 1 binding protein 1	1.02	0.02
KMT2C	Lysine (K)-specific methyltransferase 2C	1.03	0.01
LAMB1	Laminin, beta 1	1.11	0.02
MAZ	MYC-associated zinc finger protein (purine-binding transcription factor)	−1.03	0.008
MYL4	Myosin, light chain 4, alkali; atrial, embryonic	1.02	0.01
NFkB2	Nuclear factor of kappa light polypeptide gene enhancer in B-cells 2 (p52/p100)	−1.06	0.003
NOP14	NOP14 nucleolar protein	−1.08	0.008
OGG1	8-Oxoguanine DNA glycosylase	1.03	0.002
PKD2	Polycystic kidney disease 2 (autosomal dominant)	1.05	0.02
POLR2J2/POLR2J3	Polymerase (RNA) II (DNA directed) polypeptide J3	1.08	0.004
PPP4R4	Protein phosphatase 4, regulatory subunit 4	1.03	0.006
RHOH	Ras homolog family member H	−1.06	0.002
S100B	S100 calcium binding protein B	1.02	0.02
SCIN	Scinderin	1.04	0.001
STC2	Stanniocalcin 2	1.04	0.008
TAF1	TAF1 RNA polymerase II, TATA box binding protein (TBP)-associated factor, 250 kDa	1.04	0.02
TNFRSF12A	Tumor necrosis factor receptor superfamily, member 12A; TNF-like weak inducer of apoptosis (TWEAK) receptor	1.24	0.01
TNFRSF18	Tumor necrosis factor receptor superfamily, member 18	−1.02	0.005

To augment traditional pathway analyses, we evaluated the 20 genes with the largest muscle expression differences in RA and control samples (Table 5) and examined gene members of well-established skeletal muscle anabolic, catabolic, and inflammatory pathways (Table 6). The top 20 upregulated and downregulated genes by fold difference were associated with muscle remodeling, satellite cell maturation, exercise intolerance, and/or energy metabolism; for these genes, the range of differences in expression was 20–50% (Table 5). Except for NF-kB2, there was no differential expression of canonical genes involved in skeletal muscle anabolic, catabolic, or inflammatory pathways (Table 6).

**Table 5:** Genes with the greatest differences in expression between patients with rheumatoid arthritis (RA) and controls

Gene ID	Gene name and description	Fold change	*p* value
*Upregulated in RA*			
OTUD1	OUT deubiquitinase 1: removes ubiquitin molecules with probable signaling regulatory role	1.50	0.035
FEZ2^a^	Fasciculation and elongation protein zeta 2 (zygin II): reduces autophagy [32]; associated with reduced cardiorespiratory fitness [33]	1.40	0.005
PITX1^a^	Paired-like homeodomain 1: promotes muscle atrophy [34]	1.37	0.046
RNU4ATAC	RNA, U4atac small nuclear (U12-dependent splicing): codes for component of the minor spliceosome [35, 36]	1.36	0.045
ABRA^a^	Actin binding Rho activating protein: promotes myoblast differentiation and myotube maturation [24]	1.33	0.031
RCAN1^a^	Regulator of calcineurin 1: regulates fiber type patterning during differentiation	1.32	0.019
CITED2^a^	Cbp/p300-interacting transactivator, with Glu/Asp-rich carboxy-terminal domain, 2: promotes stem cell maintenance [22, 23]; prevents myofibril degradation [37]	1.32	0.027
VGLL2^a^	Vestigial-like family member 2: expressed in myotubes [27]	1.30	0.035
MYF6^a^	Myogenic factor 6 (herculin): promotes myoblast terminal differentiation [29]	1.27	0.033
RPL36AL	Ribosomal protein L36a-like: ribosomal protein with ability to terminate translation in certain situations [38]	1.27	0.011
*Downregulated in RA*			
FBP2^b^	Fructose-1,6 bisphosphatase 2: promotes glycogen storage [39, 40]; protects mitochondria from Ca2+ -induced injury [41]	−1.42	0.013
MYLK4^a^	Myosin light chain kinase family, member 4: reduced expression associated with cardiomyopathies [42]	−1.37	0.024
ZFP36^ac^	ZFP36 ring finger protein; encodes tristetraprolin (TTP): reduces inflammation and prevents satellite cell activation [20]	−1.36	0.023
DDIT4^a^	DNA damage-inducible transcript 4; also known as protein regulated in development and damage response 1 (REDD-1): promotes autophagy, with reduced expression associated with exercise intolerance [43]	−1.34	0.023
MIDN^b^	Midnolin: regulates neurogenesis [44]; reduces pancreatic glycolysis in low glucose states [45]	−1.32	0.017
SLC2A5^b^	Solute carrier family 2 (facilitated glucose/fructose transporter), member 5: performs facilitative fructose uptake into muscle [46]	−1.31	0.041
SLC25A25^b^	Solute carrier family 25 (mitochondrial carrier; phosphate carrier), member 25: promotes anti-atherosclerotic macrophage ATP production [47]; promotes muscle ATP production and physical endurance [48]	−1.30	0.013
RRAD^a^	Ras-related associated with diabetes: increases myoblast proliferation and promotes myotube formation [30]	−1.30	0.044
ZBTB16^bc^	Zinc ring finger and BTB domain containing 16: suppresses autoreactive T cells and inflammation [21]; promotes adaptive thermogenesis and mitochondrial capacity [49]	−1.27	0.050
SMTNL2	Smoothelin-like 2: associated with myotube formation [50]	−1.22	0.008

^a^Genes associated with muscle remodeling, satellite cell maturation, or exercise intolerance. See Additional file 1 for more details. ^b^Genes associated with metabolism

^c^Genes associated with immune and inflammatory responses

**Table 6:** Genes involved in skeletal muscle anabolic, catabolic, and inflammatory pathways

Gene ID	Gene name	Rheumatoid arthritis vs. control	
		Fold change	*p* value
*Ubiquitin-proteasome pathway*			
MuRF1	Muscle RING-finger protein-1	−1.02	0.25
MuRF2	Muscle-specific RING finger-2	−1.01	0.47
FbxO32	F-box protein 32	1.02	0.88
FbxO40	F-box protein 40	−1.03	0.37
*Autophagy-lysozyme pathway*			
Atg5	Autophagy related 5	−1.01	0.77
Atg7	Autophagy related 7	−1.09	0.13
NAF1	Nuclear assembly factor 1 ribonucleoprotein	−1.03	0.12
Lamp2	Lysosomal-associated membrane protein 2	−1.03	0.65
*IGF1/Akt signaling pathway*			
IGF1	Insulin-like growth factor 1	1.00	0.85
Akt1	V-Akt murine thymoma viral oncogene homolog 1	1.00	0.92
Akt2	V-Akt murine thymoma viral oncogene homolog 2	−1.04	0.41
Rptor	Regulatory associated protein of MTOR, complex 1	1.02	0.45
Rictor	RPTOR independent companion of MTOR, complex 2	1.01	0.54
FoxO1	Forkhead box O1	−1.07	0.34
FoxO3	Forkhead box O3	−1.09	0.39
*TGFbeta/Myostatin signaling pathway*			
ActRIIIB	ARP3 actin-related protein 3 homolog B	1.02	0.69
FST	Follistatin	−1.02	0.30
*NFkB signaling pathways*			
IKBKB	Inhibitor of kappa light polypeptide gene enhancer in B cells, kinase beta	−1.08	0.17
IKBKAP	Inhibitor of kappa light polypeptide gene enhancer in B cells, kinase complex-associated protein	1.001	0.43
TRAF6	TNF receptor-associated factor 6, E3 ubiquitin protein ligase	1.02	0.37
TRADD	TNFRSF1A-associated via death domain	−1.02	0.46
Bcl3	B-Cell CLL/lymphoma 3	−1.02	0.32
TRAF2	TNF receptor-associated factor 2	−1.00	0.95
TRAF5	TNF receptor-associated factor 5	1.01	0.37
MAPK8	Mitogen-activated protein kinase 8	−1.01	0.32
NFkB1	Homo sapiens nuclear factor of kappa light polypeptide gene enhancer in B cells 1 (p105/p50)	−1.00	0.97
NFkB2	Homo sapiens nuclear factor of kappa light polypeptide gene enhancer in B cells 2 (p52/p100)	−1.06	0.003

### Skeletal muscle metabolic intermediates

**Fig. 2:**
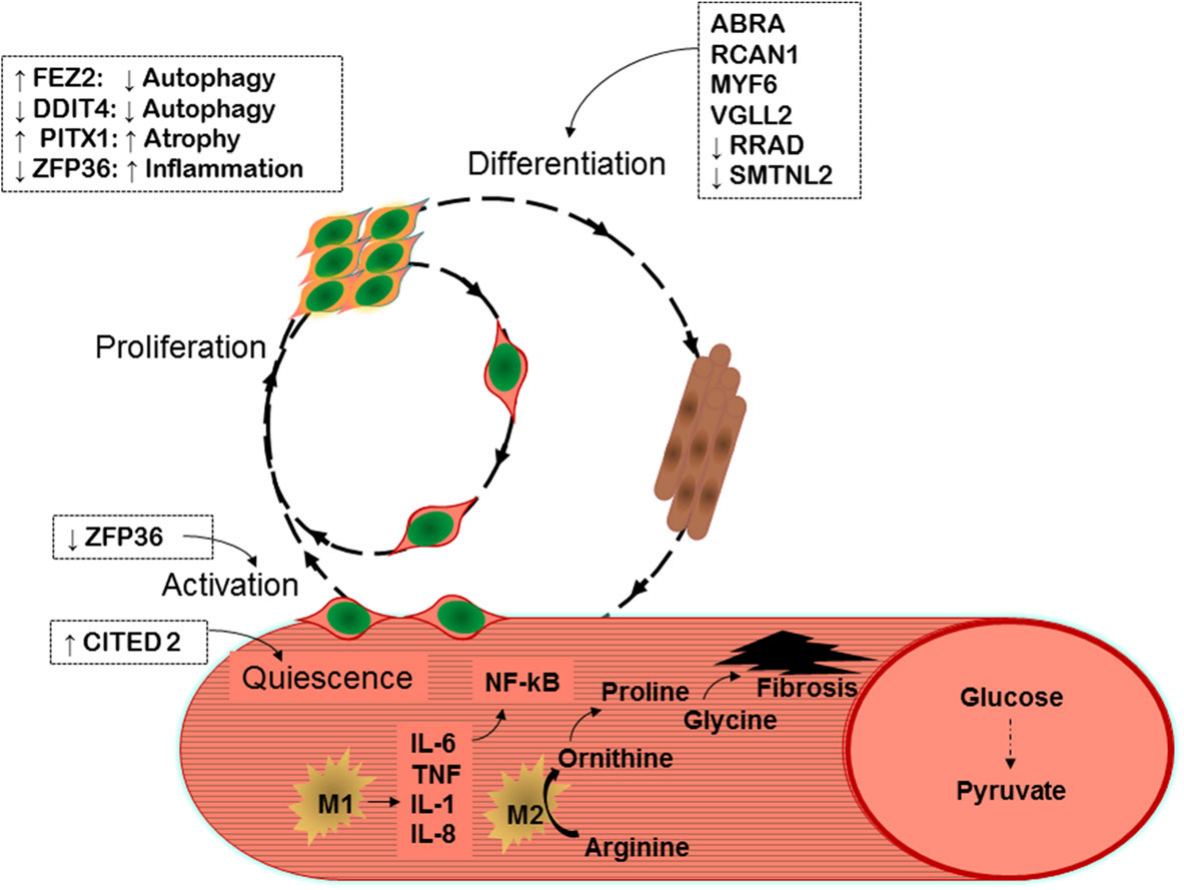
Schematic depiction of muscle injury repair showing potential impact of cytokine, gene expression, and amino acids on satellite cell activation, macrophage function, and fibrosis in muscle from patients with rheumatoid arthritis (RA). Boxes show gene IDs for genes differentially regulated in patients with RA and in controls. See Table 3 for gene descriptions

When concentrations of skeletal muscle metabolic intermediates were compared between RA (*n* = 51) and controls (*n* = 51), muscle pyruvate concentrations were 46% greater in muscle in RA than in controls (*p* < 0.001) (Table 7). There were no significant differences in the concentrations of muscle amino acids, other organic acids, or acylcarnitines in RA compared to controls (Table 7). However, several muscle amino acids and acylcarnitines were related to RA disease activity and disability. For instance, greater concentrations of glycine, serine, aspartate/asparagine, and ornithine and lower muscle concentrations of alanine and fumarate were related to greater disease activity (*p* < 0.05) (Table 8). Greater muscle concentrations of glycine, proline, ornithine, arginine, and aspartate/asparagine were related to greater disability (*p* < 0.05) (Fig. 2); in contrast, lower concentrations of several long-chain unsaturated acylcarnitines were related to greater disease activity and disability (*p* < 0.05) (Table 8).

**Table 7:** Skeletal muscle metabolic intermediate concentrations

	Rheumatoid arthririts (*n* = 51)		Controls (*n* = 50)	
	Mean	SD	Mean	SD
*Amino acids*				
Glycine	1012.669	304.568	1042.875	360.089
Alanine	2781.241	876.247	2735.464	820.802
Serine	773.987	190.246	777.726	286.455
Proline	502.861	179.5	528.023	222.984
Valine	291.82	75.004	300.739	99.003
Leucine/isoleucine	659.544	197.996	663.116	233.404
Methionine	54.167	14.36	55.372	17.48
Histidine	488.821	164.605	548.187	276.048
Phenylalanine	77.739	22.414	80.155	27.759
Tyrosine	80.962	23.249	88.815	32.052
Aspartate/asparagine	100.518	62.088	144.672	198.704
Glutamate/glutamine	2096.524	658.272	2359.04	878.482
Ornithine	212.338	85.775	184.849	69.873
Citrulline	69.446	39.718	75.05	50.441
Arginine	431.024	182.215	394.565	149.594
*Organic acids*				
Lactate	22862.683	9246.29	20956.576	8926.553
Pyruvate	1168.544*	604.675	803.474	539.098
Succinate	48.143	35.538	41.793	29.968
Fumarate	70.313	26.403	62.708	25.253
Malate	521.019	205.905	476.648	198.079
alphaKetoglutarate	144.24	143.952	113.438	118.669
Citrate	41.677	33.591	36.853	24.096
*Acylcarnitines*				
Free carnitine: C0	3369.034	1006.646	3631.978	1243.598
C2	455.175	288.39	485.702	312.966
C3	5.206	2.024	5.019	2.018
C4/Ci4	3.541	4.994	3.008	2.594
C5:1	1.033	0.397	1.03	0.41
C5	1.667	1.15	2.246	5.666
C4OH	2.789	2.231	2.378	1.778
C6	3.58	3.882	2.956	2.855
C5OH	0.676	0.363	0.65	0.343
C3DC	0.793	0.356	0.809	0.292
C4DC/Ci4DC	2.439	1.424	2.547	1.192
C8:1	0.531	0.328	0.532	0.252
C8	0.942	0.904	0.826	0.694
C5DC	1.528	1.043	1.43	0.727
C8:1OH/C6:1 DC	0.216	0.129	0.204	0.123
C6DC/C8OH	0.353	0.239	0.388	0.226
C10:3	0.067	0.047	0.067	0.034
C10:2	0.05	0.03	0.063	0.041
C10:1	0.261	0.253	0.239	0.164
C10	0.655	0.6	0.58	0.48
C7DC	0.108	0.079	0.088	0.049
C8:1 DC	0.087	0.073	0.093	0.051
C10OH:C8DC	0.305	0.253	0.31	0.21
C12:2	0.052	0.034	0.052	0.035
C12:1	0.364	0.281	0.366	0.287
C12	1.359	1.073	1.31	1.125
C12:2OH/C10:2 DC	0.075	0.045	0.064	0.04
C12:1OH/C10:1 DC	0.224	0.178	0.202	0.114
C12OH/C10DC	0.441	0.472	0.417	0.382
C14:3	0.078	0.052	0.073	0.054
C14:2	1.126	1.025	0.902	0.83
C14:1	2.726	2.354	2.449	2.232
C14	4.156	3.277	3.781	3.373
C14:3OH/C12:3 DC	0.032	0.025	0.028	0.022
C14:2OH/C12:2 DC	0.174	0.121	0.143	0.081
C14:1OH/C12:1 DC	0.704	0.538	0.701	0.431
C14OH/C12DC	0.502	0.525	0.487	0.381
C16:3	0.198	0.164	0.157	0.103
C16:2	1.533	1.201	1.199	0.948
C16:1	6.736	5.227	5.751	3.973
C16	20.041	15.253	17.878	12.497
C16:3OH/C14:3-DC	0.053	0.038	0.045	0.024
C16:2OH/C14:2 DC	0.477	0.336	0.412	0.248
C16:1OH/C14:1 DC	1.306	1.077	1.256	0.834
C16OH/C14DC	1.18	1.265	1.229	1.059
C18:3	1.463	0.982	1.354	0.925
C18:2	20.561	15.909	17.722	13.495
C18:1	46.521	37.117	40.451	28.311
C18	11.278	8.401	10.817	8.203
C18:3OH/C16:3 DC	0.186	0.158	0.168	0.101
C18:2OH/C16:2 DC	1.357	1.235	1.323	1.177
C18:1OH/C16:1 DC	2.683	2.889	2.844	2.749
C18OH/C16DC	0.695	0.68	0.732	0.523
C20:4	2.023	1.801	1.778	1.872
C20:3	0.63	0.597	0.57	0.431
C20:2	0.308	0.271	0.261	0.164
C20:1	0.554	0.484	0.485	0.409
C20	0.369	0.4	0.329	0.308
C20:3OH/C18:3 DC	0.075	0.059	0.074	0.056
C20:2OH/C18:2 DC	0.053	0.034	0.05	0.03
C20:1OH/C18:1 DC	0.071	0.062	0.062	0.046
C20OHC18DC/C22:6	0.212	0.248	0.198	0.206
C22:5	0.264	0.299	0.247	0.277
C22:4	0.241	0.279	0.193	0.158
C22:3	0.064	0.057	0.056	0.044
C22:2	0.051	0.035	0.044	0.025
C22:1	0.069	0.05	0.065	0.038
C22	0.059	0.049	0.062	0.046

Data are shown as means and standard deviations (pmol/mg tissue). Metabolic intermediates were measured in muscle homogenates. Group comparisons between muscle from patients with rheumatoid arthritis and from controls were performed using logarithmically transformed metabolic intermediates and mixed models. Prefix *C* denotes acylcarnitines followed by carbon number and degree of unsaturation. Suffixes *OH* and *DC* denote hydroxyl and dicarboxyl groups, respectively. *P < 0.05 for comparison with matched controls

**Table 8:** Relationships between rheumatoid arthritis clinical features and muscle metabolic intermediates

	Disease activity	Disability	Pain	Exercise (min/d)	Physical activity (MET h/d)
*Amino acids*					
Glycine	0.33^b^	0.50^a^	0.23	0.11	0.02
Alanine	-0.31^b^	0.03	-0.01	0.11	0.08
Serine	0.31^b^	0.20	0.17	-0.03	-0.01
Proline	0.20	0.36^a^	0.09	0.05	0.08
Valine	0.12	0.11	-0.04	0.05	-0.01
Leucine/isoleucine	0.09	0.18	-0.01	-0.08	-0.16
Methionine	0.07	0.16	-0.17	-0.01	-0.04
Histidine	-0.08	-0.02	-0.12	0.23	0.19
Phenylalanine	-0.06	-0.04	-0.21	0.06	0.04
Tyrosine	-0.06	0.08	-0.13	0.13	0.14
Aspartate/asparagine	0.34^b^	0.36^a^	0.20	-0.13	-0.12
Glutamate/glutamine	0.20	0.24	0.06	-0.12	-0.04
Ornithine	0.32^b^	0.39^a^	0.14	-0.21	-0.20
Citrulline	0.08	0.21	0.13	-0.02	0.13
Arginine	0.27	0.45^a^	0.24	-0.26	-0.27
*Organic acids*					
Lactate	-0.09	-0.18	-0.06	-0.09	-0.12
Pyruvate	-0.22	-0.22	-0.21	0.16	0.05
Succinate	0.03	-0.01	0.15	-0.06	-0.12
Fumarate	-0.34^b^	-0.24	-0.15	0.05	-0.01
Malate	-0.28	-0.14	0.04	-0.01	-0.07
alphaKetoglutarate	-0.22	-0.03	-0.03	0.27	0.18
Citrate	0.17	0.23	0.29	0.05	0.14
*Acylcarnitines*					
Free carnitine: C0	-0.10	0.19	0.10	0.04	0.08
C2	-0.07	0.08	0.07	-0.22	-0.02
C3	-0.05	0.10	-0.01	0.10	0.00
C4/Ci4	-0.02	-0.13	-0.20	0.09	0.16
C5:1	0.15	0.09	0.06	-0.10	0.05
C5	0.01	0.05	-0.24	0.14	0.10
C4OH	0.11	0.09	0.11	-0.11	-0.04
C6	0.05	-0.10	-0.24	0.30	0.29
C5OH	-0.24	0.04	0.14	0.14	0.25
C3DC	-0.17	0.13	0.03	-0.02	0.10
C4DC/Ci4DC	0.02	0.28^b^	0.05	-0.32^b^	-0.23
C8:1	-0.10	-0.05	-0.15	-0.07	0.00
C8	0.01	-0.11	-0.13	0.16	0.13
C5DC	0.19	0.20	0.03	-0.12	-0.11
C8:1OH/C6:1 DC	0.11	0.21	0.12	-0.05	-0.11
C6DC/C8OH	-0.02	0.03	-0.10	0.08	0.11
C10:3	0.02	0.15	0.15	-0.10	-0.09
C10:2	0.00	0.06	-0.05	-0.14	-0.20
C10:1	-0.09	-0.09	-0.08	0.19	0.11
C10	-0.05	-0.12	-0.13	0.15	0.14
C7DC	0.04	0.13	0.06	-0.20	-0.20
C8:1 DC	-0.17	-0.03	-0.13	-0.06	-0.09
C10OH:C8DC	-0.08	0.00	-0.15	0.06	0.07
C12:2	0.04	-0.01	-0.08	-0.24	-0.26
C12:1	-0.14	-0.12	-0.13	0.14	0.12
C12	-0.20	-0.22	-0.19	0.20	0.19
C12:2OH/C10:2 DC	-0.19	-0.03	-0.14	0.06	0.00
C12:1OH/C10:1 DC	-0.19	-0.07	-0.16	0.15	0.18
C12OH/C10DC	-0.16	0.03	-0.13	0.13	0.14
C14:3	-0.14	-0.09	-0.15	0.19	0.20
C14:2	-0.22	-0.17	-0.22	0.27	0.26
C14:1	-0.18	-0.16	-0.17	0.21	0.21
C14	-0.25	-0.21	-0.26	0.24	0.22
C14:3OH/C12:3 DC	-0.05	0.08	-0.01	-0.03	0.12
C14:2OH/C12:2 DC	-0.12	-0.03	-0.20	0.04	0.03
C14:1OH/C12:1 DC	-0.24	-0.11	-0.19	0.14	0.17
C14OH/C12DC	-0.13	0.03	-0.14	0.16	0.17
C16:3	-0.28	-0.19	-0.22	0.26	0.27
C16:2	-0.34^b^	-0.26	-0.26	0.35^a^	0.33^b^
C16:1	-0.28	-0.22	-0.17	0.22	0.18
C16	-0.27	-0.20	-0.19	0.17	0.18
C16:3OH/C14:3-DC	-0.16	-0.10	-0.03	0.11	0.19
C16:2OH/C14:2 DC	-0.26	-0.16	-0.19	0.12	0.10
C16:1OH/C14:1 DC	-0.25	-0.09	-0.21	0.10	0.12
C16OH/C14DC	-0.17	0.04	-0.08	0.10	0.12
C18:3	-0.43^a^	-0.36^a^	-0.19	0.20	0.19
C18:2	-0.40^a^	-0.39^a^	-0.19	0.23	0.18
C18:1	-0.33^b^	-0.32^b^	-0.15	0.15	0.13
C18	-0.21	-0.13	-0.12	0.06	0.09
C18:3OH/C16:3 DC	-0.29	-0.06	-0.02	0.18	0.18
C18:2OH/C16:2 DC	-0.31^b^	-0.06	-0.08	0.12	0.14
C18:1OH/C16:1 DC	-0.22	0.02	-0.04	0.06	0.07
C18OH/C16DC	-0.18	0.03	-0.08	-0.03	0.00
C20:4	-0.28	-0.30^b^	-0.12	0.26	0.27
C20:3	-0.29^b^	-0.37^a^	-0.11	0.18	0.17
C20:2	-0.25	-0.20	-0.14	0.11	0.14
C20:1	-0.25	-0.16	-0.11	0.07	0.08
C20	-0.18	-0.04	-0.09	-0.02	-0.01
C20:3OH/C18:3 DC	0.09	0.21	-0.01	-0.14	-0.08
C20:2OH/C18:2 DC	-0.16	-0.15	0.06	-0.14	-0.14
C20:1OH/C18:1 DC	-0.03	0.18	-0.03	0.00	0.04
C20OHC18DC/C22:6	-0.16	0.00	-0.09	-0.05	-0.08
C22:5	-0.28	-0.20	-0.14	0.15	0.09
C22:4	-0.22	-0.24	-0.06	0.07	0.09
C22:3	-0.03	-0.11	0.01	-0.06	-0.03
C22:2	0.13	-0.01	-0.06	0.09	0.24
C22:1	0.06	0.01	-0.12	0.28	0.36
C22	-0.02	-0.06	0.00	0.03	0.10

Data are shown as Spearman correlation coefficients. ^a^Significant relationships (*p* < 0.05) to all red and green color and ^b^Significant relationships r ≥ |0.35| to all bright red and green

## Discussion

Here, we report that in RA, skeletal muscle exhibits molecular alterations in inflammatory markers, transcriptional profiles, and metabolic signatures. Both at protein and transcriptional levels, muscle had a pro-inflammatory phenotype in RA. Additionally, differential gene expression in muscle in RA was indicative of dysregulation of muscle repair, promotion of glycolysis, and poor mitochondrial function. Upregulated glycolysis and mitochondrial inefficiency were supported by greater muscle concentrations of the glycolytic end-product pyruvate in RA. Further, disease activity and disability were related to lesser concentrations of long-chain acylcarnitines and greater concentrations of amino acid precursors for muscle fibrosis. Taken together, these alterations in proteins, gene expression, and metabolic intermediates were indicative of muscle in RA in a state of chronically activated, yet dysregulated remodeling with increased glycolysis, mitochondrial inefficiency, and fibrotic material (Fig. 2).

This represents the first report of significant markers of inflammation in muscle in RA. The clinical importance of these molecules is demonstrated by the significant association of several muscle cytokines with RA disease activity, disability, pain, and physical inactivity. The IPA-generated novel network centered on downregulation of NF-kB2, a protein that promotes non-canonical NF-kB signaling and opposes inflammatory signaling [17]. Downregulation of NF-kB2 would be predicted to favor coordinated upregulation of pro-inflammatory NF-kB signaling in muscle in RA. We were unable to determine if the muscle cytokines and pro-inflammatory transcripts in RA were derived from myocytes, inflammatory cells, or other cellular sources. Interestingly, muscle cytokine concentrations did not reflect those measured in circulation, suggesting these disease-associated inflammatory markers stem from local rather than systemic events.

Based on the strong relationships between muscle inflammatory markers and disability, pain and physical inactivity, we suspected that increased intramuscular cytokines may be indicative of a disrupted muscle remodeling process. In fact, muscle gene expression alterations in RA were consistent with promotion of satellite cell differentiation and upregulation of several facets of the normally well-coordinated process of muscle remodeling. For instance, muscle in RA was characterized by downregulation of ZFP36, the gene that encodes tristetraprolin (TTP), which reduces inflammation by destabilizing pro-inflammatory cytokine transcripts [18, 19] and prevents satellite cell activation by destabilizing myogenic regulatory factor, MyoD, mRNA [20]. Thus, the reduction in ZFP36 expression in muscle in RA would be expected to promote pro-inflammatory cytokine production and satellite cell activation.

Other gene expression changes also suggest both chronic activation and temporal dysregulation of muscle remodeling. For instance, downregulation of ZBTB16 would promote inflammation and proliferation of autoreactive T cells [21]. In contrast to the reduced ZFP36 expected to promote satellite cell activation, the increased CITED2 would be expected to reduce satellite cell activation [22, 23]. Increased expression of ABRA, RCAN1, VGLL2, MYF6 and decreased expression of RRAD would promote differentiation of satellite cells [24–30]. More descriptions of differentially expressed genes are provided in Additional file 1.

Gene expression alterations indicative of glycolysis promotion and poor mitochondrial function were supported by greater muscle concentrations of the glycolytic end-product pyruvate in RA. Further, disease activity and disability were related to lower concentrations of fatty-acid-derived long-chain acylcarnitines. One plausible explanation for this relationship is that fewer long-chain acylcarnitines indicate less oxidative metabolism and fewer mitochondria, consistent with a glycolytic phenotype. RA disease activity and disability were also related to higher concentrations of amino acid precursors for muscle fibrosis. M2-type macrophages contain arginase, which metabolizes arginine to ornithine [31]. Ornithine is converted to proline, which provides a substrate for resident fibroblasts to generate collagen. In addition to proline, collagen formation also requires glycine; glycine and proline each account for a third of the collagen amino acids. While collagen is critical for extracellular matrix production, in the setting of a chronically activated remodeling process, excess collagen production leads to fibrosis [31]. Thus, the relationships between these amino acids and disease activity and disability may indicate a fibrotic process in muscle associated with active disease that contributes to RA-associated disability.

There were several limitations to this study. RA medication regimens were not uniform among participants, and effects of these medications on skeletal muscle are unclear. Twenty-five percent of patients with RA used prednisone at low doses, which is not expected to have significant myopathic effects; despite this, they had significant alterations in muscle inflammatory markers and systemic inflammation relative to controls. Without histopathologic assessment or single cell isolations, we were unable to determine the cellular source of muscle cytokines, transcripts, or metabolites. Our findings indicate that either RA medication regimens or the RA disease process itself alters skeletal muscle inflammatory molecules, transcriptional profiles, and metabolic pathways.

## Conclusions

Taken together, these alterations in pro-inflammatory cytokines, gene expression, and metabolic intermediates are indicative of RA muscle in a state of chronically activated, yet dysregulated remodeling, with increased glycolysis, mitochondrial inefficiency, and fibrosis. It is very likely these changes contribute to the ongoing issues of exercise intolerance and disability in persons with RA. Future work should be directed at understanding whether these deficits may be mitigated by combining pharmacologic treatment with physical activity, to reduce inflammatory signaling and/or fibrosis while promoting skeletal muscle efficiency. Therefore, to improve the lives of patients with RA, future work should be directed toward understanding the role of skeletal muscle in RA, and interactions between treatment regimens, physical activity, and influences of skeletal muscle on the clinical status in RA.

## Additional file

Additional file 1: Supplemental gene information. Additional detail on genes that were most differentially expressed in muscle from patients with rheumatoid arthritis and from control participants. Genes are described and categorized by the proposed function of their respective gene products (DOCX 88 kb)

## Abbreviations

BMI: body mass index
BSA: bovine serum albumin
DAS-28: disease activity score
DMARD: disease modifying anti-rheumatic drugs
ELISA: enzyme-linked immunosorbent assay
HAQ-DI: health assessment questionnaire-disability index
HOMA: homeostasis model assessment
hsCRP: high sensitivity C-reactive protein
Hu: Houndsfield units
IL: interleukin
IPA: Ingenuity Pathway Analysis
LC: liquid chromatography
m: muscle
MET: metabolic equivalent
MS: mass spectrometry
NaN: not a number
NF-kβ: nuclear factor-kβ
NMR: nuclear magnetic resonance
PBS: phosphate-buffered saline
RA: rheumatoid arthritis
TLR: toll-like receptor
TNF-α: tumor necrosis factor-alpha
TTP: tristetraprolin
VAS: visual analog scale
