# Supplementary material for: Molecular alterations in skeletal muscle in rheumatoid arthritis are related to disease activity, physical inactivity, and disability
Source: Arthritis Res Ther. 2017 Jan 23;19:12. doi: 10.1186/s13075-016-1215-7 (PMC5260091; doi:10.1186/s13075-016-1215-7)
Supplement: Additional file 1: — Supplemental gene information. Additional detail on genes that were most differentially expressed in muscle from patients with rheumatoid arthritis and from control participants. Genes are described and categorized by the proposed function of their respective gene products (DOCX 88 kb) [file 13075_2016_1215_MOESM1_ESM.docx]

**Supplemental Gene Information:**

Below is a summary of RA skeletal muscle differential gene expression. Changes are categorized into several themes including inflammation, satellite cell activation and proliferation, muscle function, metabolism, and transcription, translation and protein degradation.

**Inflammation**

The prominent connections in top IPA novel network (novel network #1) center on downregulation of NF-kB2, a member of the NF-kB complex, which is a major regulator and activator of both innate and adaptive immune responses.  NF-kB2 promotes non-canonical NF-kB signaling and reduces inflammation [[1](#_ENREF_1)]. Tumor necrosis factor receptor superfamily, member 12a (TNFRSF12A or TWEAK receptor) was directly connected to the NF-kB complex, and was found to be significantly up-regulated. TWEAK receptor has previously been shown to be up-regulated in the context of inflammation, and potentiates pro-inflammatory responses to TNF and IL-1 [[2](#_ENREF_2)]. This finding is consistent with increased RA skeletal muscle M1-type macrophage activity. Additionally, increased muscle TWEAK receptor reduces myogenic potential and has been proposed as poor prognostic measure of post-orthopedic surgical muscle repair [[3](#_ENREF_3)]. The tumor necrosis factor receptor superfamily, member 18 (TNFRSF18, or glucocorticoid-induced TNF receptor) was found to be down-regulated. TNFRSF18 expression is increased in RA synovium [[4](#_ENREF_4)], but also indicates active regulatory T cells [[5](#_ENREF_5)], important in latter stages of muscle repair [[6](#_ENREF_6)]. Interleukin-1 receptor-associated kinase 1 binding protein 1 (IRAK1BP1) is known to inhibit inflammation by altering the balance of NFkB subunits and promoting anti-inflammatory IL-10 production [[7](#_ENREF_7)]. IRAK1BP1 expression was increased in RA muscle, while IL-10 expression was reduced 1.03 fold (P<0.01). Thus, in the setting of the aforementioned changes, increased expression of IRAK1BP1 and reduced expression of TNFRSF18 suggests RA muscle is maintained in earlier phases of muscle repair.

**Muscle remodeling, satellite cell proliferation and/or differentiation**

As implied above, several differentially expressed molecules have functions connecting muscle inflammation to muscle injury response. One of these downregulated in RA muscle was ZFP36. ZFP36 encodes tristetraprolin (TTP) which reduces inflammation by destabilizing pro-inflammatory cytokine transcripts. These cytokines include macrophage-produced TNF, as well as muscle produced-IL6, CXCL1, and CCL2 (MCP-1) [[8](#_ENREF_8), [9](#_ENREF_9)] In skeletal muscle, TTP maintains satellite cells in a primed, but quiescent state by destabilizing MyoD mRNA and preventing satellite cell activation [[10](#_ENREF_10)]. Upon injury, p38/MAPK phosphorylates and inactivates TTP, promoting satellite cell activation.[[10](#_ENREF_10)] In RA, a reduction in expression of ZFP36 or TTP would be expected to promote pro-inflammatory cytokine production. Also, reduced TTP expression may reflect a failure of the p38/MAPK pathway to phosphorylate and stabilize TTP and/or prevent inappropriate satellite cell activation.

Upregulation of the c-AMP-responsive element binding protein (CBP)/p300-interacting transactivator with ED-rich tail 2, CITED2, could have several RA muscle implications. CITED2 regulates many activities of the CREB binding protein and p300 pathways. CITED2 is critical for stem cell quiescence, is upregulated by hypoxia, and reduces cellular responses to hypoxia; these functions are likely responsible for CITED2’s role in embryonic development of multiple tissues [[11](#_ENREF_11), [12](#_ENREF_12)]. In mature cells, CITED2 represses macrophage activation of NFkB, reduces chondrocyte and osteoblast expression of matrix metalloproteinases, and increases liver expression of gluconeogenesis genes [[13-16](#_ENREF_13)]. In muscle, insulin reduces CITED 2 expression [[16](#_ENREF_16)]. Myocyte CITED2 reduces glucocorticoid-induced muscle atrophy by preventing ubiquitin proteasome-mediated degradation of myofibrillar proteins [[17](#_ENREF_17)]. Thus, upregulation in RA muscle may be a response to hypoxia or glucocorticoid exposure. Based on its role in stem cell quiescence, embryonic development, cartilage and bone remodeling, increased RA CITED2 reflects an ongoing muscle remodeling process.

Upregulated in RA, regulator of calcineurin 1 (RCAN1) regulates fiber type differentiation during myogenesis, [[18](#_ENREF_18)] increases following exhaustive exercise, and expression is augmented by oxidative stress [[19](#_ENREF_19)]. RCAN1 promotes T cell development, but excess expression is associated with immune dysfunction [[20](#_ENREF_20)].

Myogenic factor 6 (herculin, MYF6, also known as myogenic regulatory factor 4) is upregulated in skeletal muscle following acute resistance exercise, and is implicated in muscle size regulation and myogenesis terminal differentiation remodeling [[21](#_ENREF_21), [22](#_ENREF_22)]. MYF6 promotes myoblast proliferation, prevents differentiation, and promotes terminal differentiation.

ABRA (Actin binding Rho activating protein) was upregulated in RA muscle. Skeletal muscle ABRA increases after resistance training and in persons with type 2 diabetes [[23](#_ENREF_23)]. ABRA appears to link mechanical stimulation of skeletal muscle to transcription for early adaptive responses to exercise for muscle remodeling and repair, including myoblast differentiation and myotube maturation [[24](#_ENREF_24)]. Chronic overexpression drives a maladaptive muscle response such as that seen in idiopathic cardiomyopathy [[23](#_ENREF_23)].

Vestigial-like 2 (VGLL2) expression was increased in RA muscle. VGLL2 is a critical member of the coordinated process of skeletal muscle development [[25](#_ENREF_25), [26](#_ENREF_26)]. VGLL2 expression is activated by MyoD and is required for myosin heavy chain expression[[26](#_ENREF_26)]. VGLL2 is expressed in all types of developing skeletal muscle with myotube-rather than myoblast-specific function [[26](#_ENREF_26)]. Thus, increased VGLL2 expression in RA would be expected to promote RA skeletal muscle differentiation.

RRAD (Ras-related associated with diabetes), downregulated in RA muscle, produces a GTPase that inhibits voltage gated calcium channels and plays two critical roles in skeletal muscle regeneration. In early stages, six to 48 hours, after muscle injury, RRAD expression increases within muscle progenitor cells, increasing proliferation and repressing differentiation. At later stages of muscle regeneration, RRAD expression inhibits Rho/ROK signaling and promotes myotube fusion [[27](#_ENREF_27)]. This expression pattern is similar to MyoD, which promotes RRAD transcription with enhanced transcription in the presence of calcineurin [[27](#_ENREF_27)]. RRAD muscle expression is increased by insulin, which decreases expression of CITED2 [[28](#_ENREF_28)]. Since systemic insulin concentrations and insulin sensitivity are similar in RA and controls [[29](#_ENREF_29)], decreased muscle RRAD in RA suggests altered response to muscle injury.

**Muscle function**

Several differentially expressed molecules have phenotypes associated with poor muscle function. Overexpression of paired-like homeodomain 1 (PITX1) has been implicated in facioscapulohumeral muscular dystrophy. PITX1 over-expression in mice led to significant loss of body weight and muscle mass, decreased muscle strength, and reduction of muscle fiber diameters, with skeletal muscle pathology showing atrophic muscle fibers with mild necrosis and inflammatory infiltration [[30](#_ENREF_30)].

Another upregulated gene was fasciculation and elongation protein zeta 2 (FEZ2). Skeletal muscle expression of FEZ2 has been associated with older age, greater BMI, and poorer cardiorespiratory fitness (VO_2peak_) and serves as a negative regulator of autophagy [[31](#_ENREF_31), [32](#_ENREF_32)]. FEZ2 is hypothesized to play a role in morphologic changes in embryonic development [[33](#_ENREF_33)]; thus, FEZ2 may be involved in skeletal muscle remodeling.

Downregulation of gene and protein expression of MYLK4, myosin light chain kinase-4, is present in heart muscle in both ischemic and dilated cardiomyopathies [[34](#_ENREF_34)]. MYLK4 is a member of the family of myosin light chain kinases, which are critical for myosin phosphorylation and muscle contraction.

Solute carrier family 25, member 25 (SLC25A25) was also found to be downregulated comparatively in RA skeletal muscle. SLC25A25 codes for a calcium-regulated mitochondrial transporter of ATP-Mg^2+^ and P_i_ across the mitochondrial inner membrane. Reduction of muscle SLC25A5 leads to dysregulated ATP homeostasis, reduced physical endurance, and metabolic inefficiency [[35](#_ENREF_35)]. SLC25A25 assists in macrophage mitochondrial ATP production critical for cholesterol efflux and atherosclerosis prevention; this anti-atherosclerotic role is supported by reduced SLC25A25 expression in atherosclerotic plaques [[36](#_ENREF_36)]. Overall, these results suggest that in RA muscle reduced SLC25A25 contributes to inefficient metabolism and decreased endurance.

In RA muscle there was decreased expression of DNA damage-inducible transcript 4 (DDIT4). DDIT-4 expression can be increased by hypoxia, glucocorticoids, exercise, and acute alcohol ingestion. During periods of stress, DDIT-4 inhibits mTOR to downregulate protein synthesis [[37](#_ENREF_37)]. Reduced muscle expression of DDIT-4 results in failure of muscle autophagy leading to mitochondrial defects, impaired oxidative phosphorylation and ATP generation, and exercise intolerance [[38](#_ENREF_38)].

Zinc finger and BTB domain containing 16 (ZBTB16) was downregulated in RA muscle. A transcription factor with many roles, ZBTB16 expression is increased in undifferentiated hematopoetic stem cells, differentiated mesenchymal stem cells undergoing osteogenesis and chondrogenesis, and differentiated immune cells [[39](#_ENREF_39)]. While ZBTB16 function in myogensis has not been recognized, based on roles for osteogenesis and chondrogenesis, ZBTB16 expression in muscle may reflect poorer myogenesis and muscle repair. Also, ZBTB16 promotes antiviral interferon-associated expression, limits inflammation in response to infection, and reduces proliferation of autoreactive T cells [[39](#_ENREF_39)]. In brown adipocytes and skeletal muscle, ZBTB16 expression increases in response to cold exposure, in a process known as adaptive thermogenesis [[40](#_ENREF_40)]. During thermogenesis, ZBTB16 increases expression of genes involved in glycolysis, fatty acid oxidation, mitochondrial respiration, and mitochondrial biogenesis; these pathways generate energy, which is dissipated as heat via ZBTB16 induction of UCP1 [[40](#_ENREF_40)]. ZBTB16 expression is inversely related to body fat and mass [[40](#_ENREF_40)]. Thus, downregulation of ZBTB16 in RA muscle may lead to less mitochondrial potential and drive energy storage rather than utilization.

**Metabolism**

There were RA-associated reductions in expression of the metabolic regulators FBP2 (Fructose-1,6 bisphosphatase 2) and MIDN. The product of FBP2, fructose 1,6 bisphosphatase, converts fructose 1,6 bisphosphate to fructose 6-phosphate, shifting glycolytic flux towards towards glycogen storage [[41](#_ENREF_41)]. In dividing satellite cells, FBP2 is located in the nucleus, but moves to the cytoplasm with differentiation into myocytes [[42](#_ENREF_42)]. In myocytes, FBP2 associates with muscle Z-lines, forming a complex with alpha-actinin and aldolase [[43](#_ENREF_43)]. This sarcomeric complex facilitates substrate transfer for glyconeogenesis and protects FBP2 from inhibition from AMP and calcium [[44](#_ENREF_44)]. AMP inhibits uncomplexed or free FBP2, which permits glycolysis rather than a futile cycle of substrates between phosphofructokinase and FBP2 [[44](#_ENREF_44)]. At increased intra-muscular concentrations of calcium (during contraction) and with GSK3 inhibition, muscle FBP2 relocates from the Z line to associate with and protect muscle mitochondria from calcium-induced swelling [[45](#_ENREF_45)]. Thus, in RA muscle, FBP2 down-regulation would promote glycolysis, reduce glycogen storage, increase mitochondrial damage, and could indicate fewer active satellite cells and disrupted muscle remodeling.

Expression of MIDN, which produces midnolin, was downregulated in RA muscle. Midnolin was identified in mouse embryo midbrains as a nucleolar molecule thought to regulate neurogenesis [[46](#_ENREF_46)]. Midnolin contains a ubiquitin-like domain, which often participate in regulation of signal transduction [[46](#_ENREF_46), [47](#_ENREF_47)]. In pancreatic cells at low glucose concentrations, midnolin inhibits the glycolytic enzyme, glucokinase, preserving glucose from the glycolytic process [[47](#_ENREF_47)]. While muscle function of MIDN is unknown, one may consider that MIDN downregulation in RA muscle would promote glycolysis.

The SLC2A5 product, GLUT-5, is expressed predominantly in glycolytic muscle fibers and performs facilitative fructose uptake into muscle [[48](#_ENREF_48)]. In skeletal muscle, GLUT5 expression is regulated by hormones, rather than dietary changes; muscle GLUT5 expression and function is increased by chronic (24 hours) exposure to insulin [[49](#_ENREF_49)]. Muscle expression is greater in T2DM, and is reduced following pioglitazone treatment [[50](#_ENREF_50)]. Downregulation in RA muscle may reflect reduced insulin concentrations, fewer glycolytic fibers, or a shift towards promoting glucose utilization.

**Transcription, translation and protein degradation**

RNU4ATAC codes for a minor spliceosome component and mutations are associated with Microcephalic osteodysplastic primordial dwarfism type I and Roifman Syndrome, two rare genetic disorders with distinct phenotypes both involving skeletal abnormalities [[51](#_ENREF_51), [52](#_ENREF_52)]. Interesting in the context of autoimmune disease and RA, Roifman Syndrome is associated with inability to produce specific antibodies [[51](#_ENREF_51)]. Increased RNU4ATAC expression in RA muscle may be expected to increase splicing of minor-intron containing genes, but without clear effects relevant to RA.

Ribosomal protein L36a-like (RPL36AL) codes for a ribosomal protein. Among a number of ribosomal proteins RPL36AL has the unique ability to bind both rRNA and tRNA, potentially hydrolyzing peptidyl-tRNA bonds for translation termination [[53](#_ENREF_53)]. This reaction may be important in cases of stalled translation [[54](#_ENREF_54)]. Thus, upregulation in RA muscle may reflect attempts to increase protein synthesis, overcome stalled translation, and/or prematurely terminate translation.

The most upregulated (1.5 fold) gene in RA muscle was OTUD1 (or DUBA7) which codes for OTU deubiquitinase 1. OTUD1 is a member of a family of enzymes that remove ubiquitin molecules to regulate a variety of cellular and signaling processes. The effect increased OTUD1 expression in RA muscle is unclear.

**References:**

1. Cubillos-Zapata C, Hernandez-Jimenez E, Toledano V, Esteban-Burgos L, Fernandez-Ruiz I, Gomez-Pina V, Del Fresno C, Siliceo M, Prieto-Chinchina P, Perez de Diego R, et al: **NFkappaB2/p100 is a key factor for endotoxin tolerance in human monocytes: a demonstration using primary human monocytes from patients with sepsis.** *J Immunol* 2014, **193:**4195-4202. doi: 4110.4049/jimmunol.1400721. Epub 1402014 Sep 1400715.

2. Perper SJ, Browning B, Burkly LC, Weng S, Gao C, Giza K, Su L, Tarilonte L, Crowell T, Rajman L, et al: **TWEAK is a novel arthritogenic mediator.** *J Immunol* 2006, **177:**2610-2620.

3. Bamman MM, Ferrando AA, Evans RP, Stec MJ, Kelly NA, Gruenwald JM, Corrick KL, Trump JR, Singh JA: **Muscle inflammation susceptibility: a prognostic index of recovery potential after hip arthroplasty?** *Am J Physiol Endocrinol Metab* 2015, **308:**E670-679. doi: 610.1152/ajpendo.00576.02014. Epub 02015 Feb 00510.

4. Bae E, Kim WJ, Kang YM, Suk K, Koh EM, Cha HS, Ahn KS, Huh TL, Lee WH: **Glucocorticoid-induced tumour necrosis factor receptor-related protein-mediated macrophage stimulation may induce cellular adhesion and cytokine expression in rheumatoid arthritis.** *Clin Exp Immunol* 2007, **148:**410-418. Epub 2007 Mar 2015.

5. Ronchetti S, Ricci E, Petrillo MG, Cari L, Migliorati G, Nocentini G, Riccardi C: **Glucocorticoid-induced tumour necrosis factor receptor-related protein: a key marker of functional regulatory T cells.** *J Immunol Res* 2015, **2015:171520.:**10.1155/2015/171520. Epub 172015 Apr 171515.

6. Saini J, McPhee JS, Al-Dabbagh S, Stewart CE, Al-Shanti N: **Regenerative function of immune system: Modulation of muscle stem cells.** *Ageing Res Rev* 2016, **27:67-76.:**10.1016/j.arr.2016.1003.1006.

7. Conner JR, Smirnova, II, Moseman AP, Poltorak A: **IRAK1BP1 inhibits inflammation by promoting nuclear translocation of NF-kappaB p50.** *Proc Natl Acad Sci U S A* 2010, **107:**11477-11482. doi: 11410.11073/pnas.1006894107. Epub 1006892010 Jun 1006894107.

8. Geyer BC, Ben Ari S, Barbash S, Kilbourne J, Mor TS, Soreq H: **Nicotinic stimulation induces Tristetraprolin over-production and attenuates inflammation in muscle.** *Biochim Biophys Acta* 2012, **1823:**368-378. doi: 310.1016/j.bbamcr.2011.1011.1001. Epub 2011 Nov 1019.

9. Carballo E, Lai WS, Blackshear PJ: **Feedback inhibition of macrophage tumor necrosis factor-alpha production by tristetraprolin.** *Science* 1998, **281:**1001-1005.

10. Hausburg MA, Doles JD, Clement SL, Cadwallader AB, Hall MN, Blackshear PJ, Lykke-Andersen J, Olwin BB: **Post-transcriptional regulation of satellite cell quiescence by TTP-mediated mRNA decay.** *Elife* 2015, **4:e03390.:**10.7554/eLife.03390.

11. Kranc KR, Schepers H, Rodrigues NP, Bamforth S, Villadsen E, Ferry H, Bouriez-Jones T, Sigvardsson M, Bhattacharya S, Jacobsen SE, Enver T: **Cited2 is an essential regulator of adult hematopoietic stem cells.** *Cell Stem Cell* 2009, **5:**659-665. doi: 610.1016/j.stem.2009.1011.1001.

12. Bhattacharya S, Michels CL, Leung MK, Arany ZP, Kung AL, Livingston DM: **Functional role of p35srj, a novel p300/CBP binding protein, during transactivation by HIF-1.** *Genes Dev* 1999, **13:**64-75.

13. Lee JY, Taub PJ, Wang L, Clark A, Zhu LL, Maharam ER, Leong DJ, Ramcharan M, Li Z, Liu Z, et al: **Identification of CITED2 as a negative regulator of fracture healing.** *Biochem Biophys Res Commun* 2009, **387:**641-645. doi: 610.1016/j.bbrc.2009.1007.1029. Epub 2009 Jul 1014.

14. Leong DJ, Li YH, Gu XI, Sun L, Zhou Z, Nasser P, Laudier DM, Iqbal J, Majeska RJ, Schaffler MB, et al: **Physiological loading of joints prevents cartilage degradation through CITED2.** *FASEB J* 2011, **25:**182-191. doi: 110.1096/fj.1010-164277. Epub 162010 Sep 164278.

15. Lou X, Sun S, Chen W, Zhou Y, Huang Y, Liu X, Shan Y, Wang C: **Negative feedback regulation of NF-kappaB action by CITED2 in the nucleus.** *J Immunol* 2011, **186:**539-548. doi: 510.4049/jimmunol.1001650. Epub 1002010 Nov 1001622.

16. Sakai M, Matsumoto M, Tujimura T, Yongheng C, Noguchi T, Inagaki K, Inoue H, Hosooka T, Takazawa K, Kido Y, et al: **CITED2 links hormonal signaling to PGC-1alpha acetylation in the regulation of gluconeogenesis.** *Nat Med* 2012, **18:**612-617. doi: 610.1038/nm.2691.

17. Tobimatsu K, Noguchi T, Hosooka T, Sakai M, Inagaki K, Matsuki Y, Hiramatsu R, Kasuga M: **Overexpression of the transcriptional coregulator Cited2 protects against glucocorticoid-induced atrophy of C2C12 myotubes.** *Biochem Biophys Res Commun* 2009, **378:**399-403. doi: 310.1016/j.bbrc.2008.1011.1062. Epub 2008 Nov 1024.

18. Oh M, Rybkin, II, Copeland V, Czubryt MP, Shelton JM, van Rooij E, Richardson JA, Hill JA, De Windt LJ, Bassel-Duby R, et al: **Calcineurin is necessary for the maintenance but not embryonic development of slow muscle fibers.** *Mol Cell Biol* 2005, **25:**6629-6638.

19. Emrani R, Rebillard A, Lefeuvre L, Gratas-Delamarche A, Davies KJ, Cillard J: **The calcineurin antagonist RCAN1-4 is induced by exhaustive exercise in rat skeletal muscle.** *Free Radic Biol Med* 2015, **87:290-9.:**10.1016/j.freeradbiomed.2015.1006.1023. Epub 2015 Jun 1026.

20. Martin KR, Layton D, Seach N, Corlett A, Barallobre MJ, Arbones ML, Boyd RL, Scott B, Pritchard MA: **Upregulation of RCAN1 causes Down syndrome-like immune dysfunction.** *J Med Genet* 2013, **50:**444-454. doi: 410.1136/jmedgenet-2013-101522. Epub 102013 May 101523.

21. Yang Y, Creer A, Jemiolo B, Trappe S: **Time course of myogenic and metabolic gene expression in response to acute exercise in human skeletal muscle.** *1985)* 2005, **98:**1745-1752. Epub 2004 Dec 1723.

22. Ma Z, Sun X, Xu D, Xiong Y, Zuo B: **MicroRNA, miR-374b, directly targets Myf6 and negatively regulates C2C12 myoblasts differentiation.** *Biochem Biophys Res Commun* 2015, **467:**670-675. doi: 610.1016/j.bbrc.2015.1010.1086. Epub 2015 Oct 1021.

23. Wallace MA, Lamon S, Russell AP: **The regulation and function of the striated muscle activator of rho signaling (STARS) protein.** *Front Physiol* 2012, **3:469.:**10.3389/fphys.2012.00469. eCollection 02012.

24. Lamon S, Wallace MA, Russell AP: **The STARS signaling pathway: a key regulator of skeletal muscle function.** *Pflugers Arch* 2014, **466:**1659-1671. doi: 1610.1007/s00424-00014-01475-00425. Epub 02014 Feb 00421.

25. Maeda T, Chapman DL, Stewart AF: **Mammalian vestigial-like 2, a cofactor of TEF-1 and MEF2 transcription factors that promotes skeletal muscle differentiation.** *J Biol Chem* 2002, **277:**48889-48898. Epub 42002 Oct 48889.

26. Bonnet A, Dai F, Brand-Saberi B, Duprez D: **Vestigial-like 2 acts downstream of MyoD activation and is associated with skeletal muscle differentiation in chick myogenesis.** *Mech Dev* 2010, **127:**120-136. doi: 110.1016/j.mod.2009.1010.1001. Epub 2009 Oct 1013.

27. Hawke TJ, Kanatous SB, Martin CM, Goetsch SC, Garry DJ: **Rad is temporally regulated within myogenic progenitor cells during skeletal muscle regeneration.** *Am J Physiol Cell Physiol* 2006, **290:**C379-387. Epub 2005 Oct 2012.

28. Coletta DK, Balas B, Chavez AO, Baig M, Abdul-Ghani M, Kashyap SR, Folli F, Tripathy D, Mandarino LJ, Cornell JE, et al: **Effect of acute physiological hyperinsulinemia on gene expression in human skeletal muscle in vivo.** *Am J Physiol Endocrinol Metab* 2008, **294:**E910-917. doi: 910.1152/ajpendo.00607.02007. Epub 02008 Mar 00611.

29. AbouAssi H, Tune KN, Gilmore B, Bateman LA, McDaniel G, Muehlbauer M, Huebner JL, Hoenig HM, Kraus VB, St Clair EW, et al: **Adipose depots, not disease-related factors, account for skeletal muscle insulin sensitivity in established and treated rheumatoid arthritis.** *J Rheumatol* 2014, **41:**1974-1979. doi: 1910.3899/jrheum.140224. Epub 142014 Jul 140221.

30. Pandey SN, Cabotage J, Shi R, Dixit M, Sutherland M, Liu J, Muger S, Harper SQ, Nagaraju K, Chen YW: **Conditional over-expression of PITX1 causes skeletal muscle dystrophy in mice.** *Biol Open* 2012, **1:**629-639. Epub 2012 May 2025.

31. Su J, Ekman C, Oskolkov N, Lahti L, Strom K, Brazma A, Groop L, Rung J, Hansson O: **A novel atlas of gene expression in human skeletal muscle reveals molecular changes associated with aging.** *Skelet Muscle* 2015, **5:35.:**10.1186/s13395-13015-10059-13391. eCollection 12015.

32. Spang N, Feldmann A, Huesmann H, Bekbulat F, Schmitt V, Hiebel C, Koziollek-Drechsler I, Clement AM, Moosmann B, Jung J, et al: **RAB3GAP1 and RAB3GAP2 modulate basal and rapamycin-induced autophagy.** *Autophagy* 2014, **10:**2297-2309. doi: 2210.4161/15548627.15542014.15994359.

33. Fujita T, Ikuta J, Hamada J, Okajima T, Tatematsu K, Tanizawa K, Kuroda S: **Identification of a tissue-non-specific homologue of axonal fasciculation and elongation protein zeta-1.** *Biochem Biophys Res Commun* 2004, **313:**738-744.

34. Herrer I, Rosello-Lleti E, Rivera M, Molina-Navarro MM, Tarazon E, Ortega A, Martinez-Dolz L, Trivino JC, Lago F, Gonzalez-Juanatey JR, et al: **RNA-sequencing analysis reveals new alterations in cardiomyocyte cytoskeletal genes in patients with heart failure.** *Lab Invest* 2014, **94:**645-653. doi: 610.1038/labinvest.2014.1054. Epub 2014 Apr 1037.

35. Anunciado-Koza RP, Zhang J, Ukropec J, Bajpeyi S, Koza RA, Rogers RC, Cefalu WT, Mynatt RL, Kozak LP: **Inactivation of the mitochondrial carrier SLC25A25 (ATP-Mg2+/Pi transporter) reduces physical endurance and metabolic efficiency in mice.** *J Biol Chem* 2011, **286:**11659-11671. doi: 11610.11074/jbc.M11110.203000. Epub 202011 Feb 203004.

36. Karunakaran D, Thrush AB, Nguyen MA, Richards L, Geoffrion M, Singaravelu R, Ramphos E, Shangari P, Ouimet M, Pezacki JP, et al: **Macrophage Mitochondrial Energy Status Regulates Cholesterol Efflux and Is Enhanced by Anti-miR33 in Atherosclerosis.** *Circ Res* 2015, **117:**266-278. doi: 210.1161/CIRCRESAHA.1117.305624. Epub 302015 May 305622.

37. Miyazaki M, Esser KA: **Cellular mechanisms regulating protein synthesis and skeletal muscle hypertrophy in animals.** *1985)* 2009, **106:**1367-1373. doi: 1310.1152/japplphysiol.91355.92008. Epub 92008 Nov 91326.

38. Qiao S, Dennis M, Song X, Vadysirisack DD, Salunke D, Nash Z, Yang Z, Liesa M, Yoshioka J, Matsuzawa S, et al: **A REDD1/TXNIP pro-oxidant complex regulates ATG4B activity to control stress-induced autophagy and sustain exercise capacity.** *Nat Commun* 2015, **6:7014.:**10.1038/ncomms8014.

39. Liu TM, Lee EH, Lim B, Shyh-Chang N: **Concise Review: Balancing Stem Cell Self-Renewal and Differentiation with PLZF.** *Stem Cells* 2016, **34:**277-287. doi: 210.1002/stem.2270. Epub 2016 Jan 1005.

40. Plaisier CL, Bennett BJ, He A, Guan B, Lusis AJ, Reue K, Vergnes L: **Zbtb16 has a role in brown adipocyte bioenergetics.** *Nutr Diabetes* 2012, **2:e46.:**10.1038/nutd.2012.1021.

41. Tillmann H, Eschrich K: **Isolation and characterization of an allelic cDNA for human muscle fructose-1,6-bisphosphatase.** *Gene* 1998, **212:**295-304.

42. Gizak A, Wrobel E, Moraczewski J, Dzugaj A: **Changes in subcellular localization of fructose 1,6-bisphosphatase during differentiation of isolated muscle satellite cells.** *FEBS Lett* 2006, **580:**4042-4046. Epub 2006 Jun 4027.

43. Rakus D, Pasek M, Krotkiewski H, Dzugaj A: **Interaction between muscle aldolase and muscle fructose 1,6-bisphosphatase results in the substrate channeling.** *Biochemistry* 2004, **43:**14948-14957.

44. Rakus D, Maciaszczyk E, Wawrzycka D, Ulaszewski S, Eschrich K, Dzugaj A: **The origin of the high sensitivity of muscle fructose 1,6-bisphosphatase towards AMP.** *FEBS Lett* 2005, **579:**5577-5581. Epub 2005 Sep 5528.

45. Pirog M, Gizak A, Rakus D: **Changes in quaternary structure of muscle fructose-1,6-bisphosphatase regulate affinity of the enzyme to mitochondria.** *Int J Biochem Cell Biol* 2014, **48:55-9.:**10.1016/j.biocel.2013.1012.1015. Epub 2014 Jan 1018.

46. Tsukahara M, Suemori H, Noguchi S, Ji ZS, Tsunoo H: **Novel nucleolar protein, midnolin, is expressed in the mesencephalon during mouse development.** *Gene* 2000, **254:**45-55.

47. Hofmeister-Brix A, Kollmann K, Langer S, Schultz J, Lenzen S, Baltrusch S: **Identification of the ubiquitin-like domain of midnolin as a new glucokinase interaction partner.** *J Biol Chem* 2013, **288:**35824-35839. doi: 35810.31074/jbc.M35113.526632. Epub 522013 Nov 526631.

48. Stuart CA, Yin D, Howell ME, Dykes RJ, Laffan JJ, Ferrando AA: **Hexose transporter mRNAs for GLUT4, GLUT5, and GLUT12 predominate in human muscle.** *Am J Physiol Endocrinol Metab* 2006, **291:**E1067-1073. Epub 2006 Jun 1027.

49. Douard V, Ferraris RP: **Regulation of the fructose transporter GLUT5 in health and disease.** *Am J Physiol Endocrinol Metab* 2008, **295:**E227-237. doi: 210.1152/ajpendo.90245.92008. Epub 92008 Apr 90248.

50. Stuart CA, Howell ME, Yin D: **Overexpression of GLUT5 in diabetic muscle is reversed by pioglitazone.** *Diabetes Care* 2007, **30:**925-931. Epub 2007 Jan 2024.

51. Merico D, Roifman M, Braunschweig U, Yuen RK, Alexandrova R, Bates A, Reid B, Nalpathamkalam T, Wang Z, Thiruvahindrapuram B, et al: **Compound heterozygous mutations in the noncoding RNU4ATAC cause Roifman Syndrome by disrupting minor intron splicing.** *Nat Commun* 2015, **6:8718.:**10.1038/ncomms9718.

52. Nagy R, Wang H, Albrecht B, Wieczorek D, Gillessen-Kaesbach G, Haan E, Meinecke P, de la Chapelle A, Westman JA: **Microcephalic osteodysplastic primordial dwarfism type I with biallelic mutations in the RNU4ATAC gene.** *Clin Genet* 2012, **82:**140-146. doi: 110.1111/j.1399-0004.2011.01756.x. Epub 02011 Aug 01728.

53. Hountondji C, Bulygin K, Crechet JB, Woisard A, Tuffery P, Nakayama J, Frolova L, Nierhaus KH, Karpova G, Baouz S: **The CCA-end of P-tRNA Contacts Both the Human RPL36AL and the A-site Bound Translation Termination Factor eRF1 at the Peptidyl Transferase Center of the Human 80S Ribosome.** *Open Biochem J* 2014, **8:52-67.:**10.2174/1874091X01408010052. eCollection 01408012014.

54. Cao J, Geballe AP: **Ribosomal release without peptidyl tRNA hydrolysis at translation termination in a eukaryotic system.** *RNA* 1998, **4:**181-188.
